# Multiparametric Radiogenomic Model to Predict Survival in Patients with Glioblastoma

**DOI:** 10.3390/cancers16030589

**Published:** 2024-01-30

**Authors:** Keon Mahmoudi, Daniel H. Kim, Elham Tavakkol, Shingo Kihira, Adam Bauer, Nadejda Tsankova, Fahad Khan, Adilia Hormigo, Vivek Yedavalli, Kambiz Nael

**Affiliations:** 1 Department of Radiological Sciences, David Geffen School of Medicine at University of California, Los Angeles, CA 90095, USA; 2 Department of Radiology, Kaiser Permanente Fontana Medical Center, Fontana, CA 92335, USA; 3 Department of Pathology, Icahn School of Medicine at Mount Sinai, New York, NY 10029, USA; 4 Department of Pathology, Washington University School of Medicine in St. Louis, St. Louis, MO 63110, USA; 5 Department of Oncology, Montefiore Einstein Cancer Center, Albert Einstein College of Medicine, Bronx, NY 10461, USA; 6 Department of Radiology and Radiological Science, Johns Hopkins Bayview Medical Center, Baltimore, MD 21224, USA

**Keywords:** multiparametric, radiogenomics, texture analysis, glioma, tumor segmentation, MRI, MGMT

## Abstract

**Simple Summary:**

The prognosis for patients with glioblastoma (GBM) remains dismal despite advances in tumor detection and treatment. The aim of our retrospective study was to develop a multiparametric model incorporating baseline MRI imaging, histopathological biomarkers, and demographic data to predict prognosis in patients with GBM. A training model was developed with 116 patients and validated with an external cohort of 40 patients. Three significant variables were identified as independent predictors of survival ≥ 18 months: radiomics, age, and *MGMT* status.

**Abstract:**

Background: Clinical, histopathological, and imaging variables have been associated with prognosis in patients with glioblastoma (GBM). We aimed to develop a multiparametric radiogenomic model incorporating MRI texture features, demographic data, and histopathological tumor biomarkers to predict prognosis in patients with GBM. Methods: In this retrospective study, patients were included if they had confirmed diagnosis of GBM with histopathological biomarkers and pre-operative MRI. Tumor segmentation was performed, and texture features were extracted to develop a predictive radiomic model of survival (<18 months vs. ≥18 months) using multivariate analysis and Least Absolute Shrinkage and Selection Operator (LASSO) regularization to reduce the risk of overfitting. This radiomic model in combination with clinical and histopathological data was inserted into a backward stepwise logistic regression model to assess survival. The diagnostic performance of this model was reported for the training and external validation sets. Results: A total of 116 patients were included for model development and 40 patients for external testing validation. The diagnostic performance (AUC/sensitivity/specificity) of the radiomic model generated from seven texture features in determination of ≥18 months survival was 0.71/69.0/70.3. Three variables remained as independent predictors of survival, including radiomics (*p* = 0.004), age (*p* = 0.039), and *MGMT* status (*p* = 0.025). This model yielded diagnostic performance (AUC/sensitivity/specificity) of 0.77/81.0/66.0 (training) and 0.89/100/78.6 (testing) in determination of survival ≥ 18 months. Conclusions: Results show that our radiogenomic model generated from radiomic features at baseline MRI, age, and *MGMT* status can predict survival ≥ 18 months in patients with GBM.

## 1. Introduction

Gliomas are the most common primary brain tumor in adults [1,2]. IDH-wildtype Glioblastoma (GBM), a World Health Organization (WHO) grade 4 brain tumor, accounts for approximately one half of all gliomas [3]. The standard of care treatment for GBM includes maximal resection and combination of radiation and chemotherapy, followed by chemotherapy [4]. Despite advances in detection and treatment, the prognosis of patients with GBM remains dismal, with a median survival of just 14 months [5,6], a 1-year survival of approximately 43%, and a 5-year survival of approximately 5% [3]. Unfortunately, even with early intervention, complete remission of GBM is uncommon as recurrence is inevitable.

A growing number of clinical, imaging, and histopathological biomarkers have been identified that carry diagnostic and prognostic significance in patients with GBM [7,8]. Amongst various clinical parameters, one of the most consistently recognized prognostic factors is younger age [8,9,10,11]. A prospective, multi-institution study by Weller et al. found that age < 60 is significantly (*p* < 0.001) associated with survival in patients with treated GBM [10]. Similarly, in a more recent retrospective study of newly diagnosed GBM, Stark et al. reported that age < 61 years was significantly (*p* < 0.001) associated with survival [8].

Several histopathological biomarkers have also been shown as strong predictors of survival. *IDH1* mutation has been established as an independent positive prognostic biomarker associated with significantly longer survival compared to *IDH1* wildtype [11]. This in fact has revolutionized GBM prognostication and ultimately led to the WHO incorporating biomarker status into its tumor classification system for the first time in 2016 with the introduction of *IDH* status [12]. In 2021, the WHO classification of central nervous system tumors was further modified to define GBM as *IDH*-wildtype, WHO grade 4, whereas WHO grade 4 tumors with *IDH* mutation were reclassified as astrocytomas [13]. Other favorable histopathological biomarkers associated with longer survival in patients with GBM include *ATRX* mutation [14,15], methylation of *MGMT* gene [16,17], and absence of *EGFR* signal amplification [18,19].

The heterogeneous histology of GBM increases the risk of non-representative tumor sampling for the purposes of histological assessment [20]. Furthermore, GBM has been shown to be prone to histological error on biopsy [21,22,23,24]. For example, in one retrospective study of 33 patients with primary brain lesion undergoing stereotactic biopsy followed by open resection, there was a 33% rate of discordance (either: identical cell type but different grading, different cell type but identical grading, or different cell type and different grading) [24].

For these reasons, there has been a recent impetus to further assess multimodal data available from clinical factors, histopathology, and imaging to improve prognostication in patients with GBM. The field of radiomics has emerged for the quantitative assessment of rich imaging datasets that can be used alone or in conjunction with variable genetic datapoints to predict survival in patients with GBM with some success [25,26,27]. Despite the promising results of numerous radiogenomic models in the prediction of survival in patients with GBM, a large number of these studies lack robust analysis and validation against external testing cohorts which limits their application for broad clinical use [25,26].

In this study, we aimed to assess the diagnostic performance of baseline MRI features in addition to clinical, demographic, and histopathological data for the prediction of survival of patients with GBM. Specifically, in a cohort of *IDH*-wildtype GBM patients per the WHO 2021 classification, we (1) constructed a radiomic model from preoperative MRI and evaluated its diagnostic performance in prediction of ≥18 month survival, (2) constructed a combined radiogenomics model to predict ≥ 18 month survival and assessed its added diagnostic value to radiomics, and (3) validated the diagnostic performance of this combined model in an external cohort from an outside institution.

## 2. Methods

This retrospective study was approved by an institutional review board with a waiver of informed consent. A total of 226 patients were reviewed with initial diagnosis of GBM between January 2015 and May 2020. Patients were included if they had (1) confirmed diagnosis of *IDH*-wildtype GBM by surgical resection and completed standardized treatment including radiation therapy and temozolomide chemotherapy; (2) preoperative MRI including T1 post contrast, FLAIR, and diffusion sequences; (3) available histopathological biomarkers including *IDH-1*, *EGFR*, *MGMT*, and *ATRX*; and (4) had survival data including time from diagnosis to death or time to 1st recurrence following standard treatment, whichever was first.

Patients were excluded if they did not have pre-treatment MRI (prior surgery or treatment, *n* = 27), insufficient MR image quality (*n* = 2), absence of MRI FLAIR or DWI sequences necessary for texture analysis (*n* = 48), or incomplete histological data (*n* = 33). Demographic data including sex, age, histopathological biomarkers, and survival time were documented for each patient. Survival data were obtained from the electronic medical record and dichotomized (<18 months, ≥18 months).

An external validation cohort (*n* = 40) was also included from an outside institution under an approved institutional review board for the purpose of increasing study validity and generalizability. This external cohort has identical inclusion and exclusion criteria.

### 2.1. Histopathological Data

Tumor tissue samples were obtained through routine standard of care practices from patients undergoing targeted tissue biopsy or tumor resection. Immunohistochemistry was used to determine *IDH* status (specifically *IDH1^R132H^* immunoreactivity) and *ATRX* nuclear staining on formalin-fixed paraffin embedded (FFPE) 6-micron tissue slices. Next-generation sequencing (NGS) was performed to confirm mutational status and, in rare cases, identified alternative *IDH* mutations that were not picked up by IHC. Both *IDH* and *ATRX* results were classified as wildtype vs. mutated, and only *IDH*-wildtype tumors were included for analysis. Signal amplification of *EGFR* was detected with chromogenic in situ hybridization on 6-micron FFPE tumor tissue slice. The results were classified as amplified vs. non-amplified. Pyrosequencing assays of bisulfite-treated genomic DNA were used to assess for *MGMT* methylation status, and the results were classified as methylated vs. unmethylated.

### 2.2. Image Analysis

Image acquisition was performed using a standardized preoperative brain tumor MRI protocol in accordance with consensus recommendations for a standardized brain tumor imaging protocol in clinical trials [28]. A combination of 1.5T and 3T MR scanners was used.

**Figure 1:**
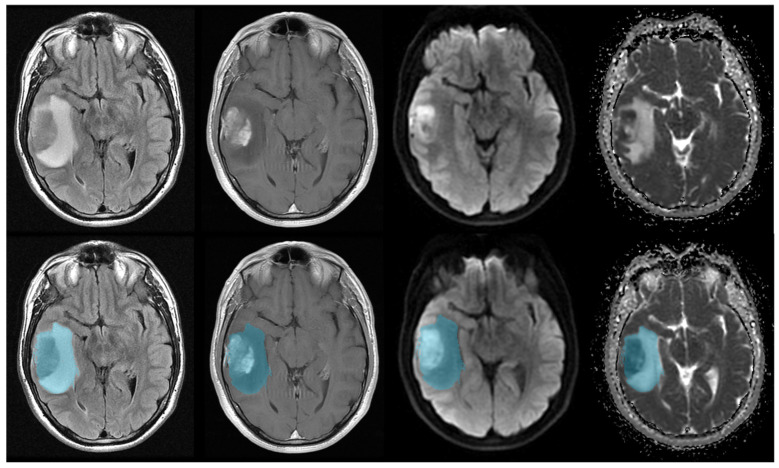
Manual segmentation of GBM in a representative patient. **Top** row: multisequence images (**left** to **right**: FLAIR, T1 post contrast, b1000, and ADC) through a single slice show GBM in the right temporal lobe. **Bottom** row: the tumor and surrounding T2 prolongation was manually segmented (shown in blue) on FLAIR images and overlaid onto other coregistered sequences.

Image analysis was performed using a commercially available FDA-approved software (Olea Sphere software version SP23.0, Olea Medical S.A.S., La Ciotat, France). FLAIR, T1 post contrast, and diffusion images (ADC/b1000) were coregistered for each patient’s pre-treatment MRI using a 6-df transformation and mutual information cost function. Tumors were manually segmented by a trained radiologist under the supervision of a board-certified neuroradiologist using FLAIR images on every slice in which tumor was visible. Subsequently, a volume of interest (VOI) was generated encompassing the entire region of FLAIR hyperintensity and overlaid onto coregistered T1 post contrast and diffusion datasets for radiomic texture analysis (Figure 1). Automatic preprocessing was standardized by the software for each case involving intensity normalization, resampling, and discretization to mitigate image signal variability and improve generalizability.

A total of 92 radiomic features were extracted from each MR sequence, including 19 first-order metrics (e.g., mean, standard deviation, skewness, and kurtosis). Additionally, numerous second-order metrics were extracted including 23 gray-level run-length matrices (GLCM), 16 gray-level run-length matrices (GLRLM), 15 gray-level size-zone matrices (GLSZM), 5 neighboring gray tone difference matrices (NGTDM), and 14 gray-level dependence matrices (GLDM). Details of the definitions and calculations of these features have been reported in the literature [29,30]. These radiomic features extracted from each patient’s pre-treatment FLAIR, T1 post contrast, and ADC/b1000 MRI sequences yielded a total of 368 features for each brain tumor.

### 2.3. Statistical Analysis

One-way analysis of variance was performed for each radiomic parameter (*n* = 368) with survival (<18 months, ≥18 months) as the independent variable. Least Absolute Shrinkage and Selection Operator (LASSO) regularization [31] was used for the statistically significant texture feature means (*p* < 0.05) to reduce the risk of overfitting and increase interpretation. The significant contributing variables were then entered into a stepdown logistic regression analysis. A stepwise method was used to avoid collinearity since redundant variables were omitted. From the independent significant radiomic variables, a final combined model was generated and validated by a 10-fold cross-validation scheme, where the data were randomly assigned into the training cohort (90%) or used for validation (10%). This final imaging model was then evaluated in conjunction with other variables including age, sex, and histopathological biomarkers in a multivariate logistic regression analysis to identify the independent predictors of survival.

Receiver-operating characteristic (ROC) curves were generated and area under the curve (AUC) was estimated for independent variables that survived logistic regression analysis. A comparative analysis between ROCs was performed using the Delong test [32]. Optimal thresholds were determined to maximize sensitivity and specificity for each biomarker utilizing the Youden index. Once the final model was constructed, it was applied to a testing dataset to determine its accuracy in predicting patient survival. Statistical analysis was performed using Matlab R2019b, Statistics and Machine Learning Toolbox (The MathWorks, Inc., Natick, MA, USA), and SAS 9.4M6 (TS1M6) 2020 (SAS Institute Inc., Cary, NC, USA).

## 3. Results

### 3.1. Clinical Characteristics of Patient Population

A total of 116 patients were included in our training dataset for model development. The survival length was 12 (5–23) [median, IQR] months. A total of 42 patients had survival of ≥18 months, whereas 74 had < 18 months survival. Patients with longer survival were significantly (*p* = 0.030) younger at the time of diagnosis. Among the histopathological biomarkers evaluated, methylated *MGMT* status was significantly associated with longer survival (*p* = 0.035). The breakdown of patients’ demographic and histopathological biomarkers are detailed in Table 1.

### 3.2. Training Model

**Figure 2:**
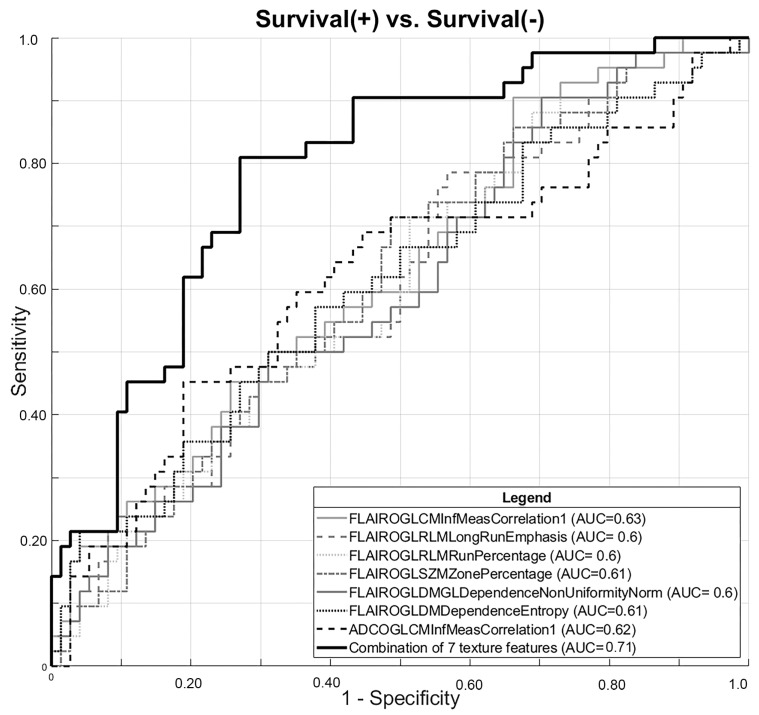
ROC curves for texture features that were identified as significant contributors for survival prediction individually and final combined radiomic model constructed from the combination of these 7 texture features.

Following LASSO regularization and logistic regression analysis, a total of 7 MRI texture features of the initial 368 remained as significant contributors in prediction of survival. The AUC attributed to each individual texture feature ranged between 0.60 and 0.63 (Supplementary Table S1). A final radiomic model was constructed from the combination of these seven features with a diagnostic performance (AUC/sensitivity/specificity) of 0.71/69.0/70.3 (*p* < 0.001). ROC curves for individual radiomic features and the final combined radiomic model are shown in Figure 2.

Following logistic regression analysis, the final radiomic model remained an independent and significant (*p* = 0.004) contributor in prediction of survival ≥ 18 months in conjunction with patient’s age (*p* = 0.039) and *MGMT* status (*p* = 0.025). The combined radiogenomic model constructed from age, MGMT status, and radiomic model showed diagnostic performances (AUC/sensitivity/specificity) of 0.77/81.0/66.0 (*p* < 0.001) in prediction of survival ≥ 18 months.

**Figure 3:**
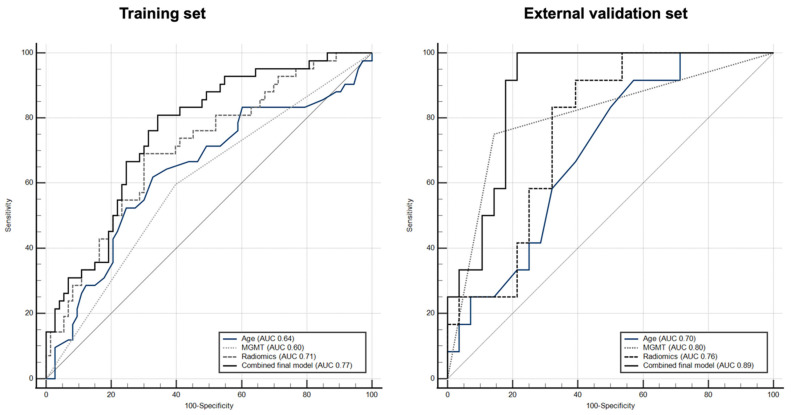
ROC curves for independent variables including age, *MGMT* status, radiomic model, and combined final radiogenomic model in training and external validation datasets.

Comparative ROC analysis between the radiogenomic model vs. individual contributors showed significantly higher diagnostic performance against the individual components using Delong test; *p* = 0.036 vs. age, *p* = 0.004 vs. *MGMT* status, and *p* = 0.044 vs. radiomic model. ROC curves for age, *MGMT* status, radiomic model, and the combined radiogenomic model in the prediction of survival ≥ 18 months are shown in Figure 3. The diagnostic accuracies of each contributing variable are listed in Table 2.

### 3.3. External Validation

External validation was performed in a cohort of 40 patients from an outside institution. In this cohort, patients had an average age of 57.2 ± 13.6 (mean ± SD) years, and 18 patients were male. The *MGMT* status was methylated in 13 patients and non-methylated in 27. A total of 12 patients had survival of ≥18 months, whereas 28 patients had < 18 months survival. The final radiogenomic model used in the training dataset showed a diagnostic performance (AUC/sensitivity/specificity) of 0.89/100/78.6 in the external validation set. ROC curves and diagnostic accuracies for age, *MGMT* status, radiomic model, and combined radiogenomic model in prediction of survival ≥ 18 months are shown for the external validation set in Figure 3 and Table 2, respectively.

## 4. Discussion

Results show that our multiparametric radiogenomic model incorporating patient’s age, texture features from baseline MRI, and *MGMT* methylation status can predict longer survival (≥18 months) in patients with IDH-wildtype, WHO grade 4 GBM at the time of diagnosis with an accuracy of 73.5% in the training cohort and 89.3% in the external validation cohort.

Younger patients’ age has been established as an important and independent prognostic factor in prediction of long-term survival [8,9,10,11]. Similarly, our results showed that younger age was significantly and independently associated with survival > 18 months. Using ROC analysis, an age cutoff of 59 years or younger was significantly associated with longer overall survival. We also showed the synergistic effect of age and *MGMT* methylation status in our final model with added predictive performance. Similarly, Hartmann et al. reported that a combination of age < 50-years-old and *MGMT* methylation can predict GBM survival > 36 months [33].

Radiomic models have shown promise in prognostication and determination of survival in patients with GBM [34,35,36,37,38,39]. In a retrospective study of 32 patients with GBM, regional MRI habitat variations were found within each tumor (e.g., high vs. low contrast enhancement; high, intermediate, and low interstitial edema) that were statistically significant in predicting patient survival with an accuracy of approximately 81% [34]. In a separate retrospective study of 82 patients with GBM, five sets of texture features were used to reliably predict 12-month survival and GBM molecular subtype with AUC of up to 0.82 [40]; some of these subtypes were more reliably predicted on FLAIR images, whereas for others, T1 post contrast sequences were more reliable. Interestingly, they found that different image planes (e.g., coronal vs. axial) had varying levels of predictive performance; however, the generalizability of these findings is limited as an independent testing cohort was not utilized. In another retrospective study of 59 patients with GBM, Drabycz et al. used both qualitative and quantitative analyses to determine whether *MGMT* promoter methylation could be predicted with various MRI sequences [41]; they found that qualitative MRI analysis showed an association between unmethylated *MGMT* status and ring enhancement, however there was no statistically significant relationship on either FLAIR or T1 post contrast images when a quantitative approach was tested. Significant quantitative differences within tumor subsections were identified on T2 weighted images, but they did not persist when the entire tumor volume was analyzed [41].

In our study, after comprehensive analysis of 368 MRI texture features obtained from FLAIR, T1 post contrast, and diffusion images (ADC/b1000), a total of seven features remained as statistically significant contributors in survival prediction (Supplementary Table S1). Chaddad et al. previously reported the prognostic value of three GLCM texture features after the analysis of 22 MRI texture features, of which one texture feature (GLCM informational measure of correlation) was also supported in our dataset [36]. Their model’s overall sensitivity using data from 40 patients with GBM was reported as 91.7%, which was higher than our training model’s sensitivity but lower than our external cohort’s. The prognostic value of GLSZM texture features is also in line with previous literature; for example, Li et al. described a fully automatic radiomics model in which a four-feature radiomics signature was identified and validated with an independent cohort to successfully stratify patients with GBM into prognostically high- and low-risk groups [37]. Although the seven features identified in our study individually predicted ≥ 18 month survival with significance, the combination of these features further improved the predictive power of our radiomic model (Figure 2).

In our study, among commonly evaluated histopathological biomarkers used in the evaluation of GBM survival, only *MGMT* methylation was found to have prognostic significance with a modest AUC (0.60). Additionally, *MGMT* methylation was significant (*p* = 0.035) in predicting survival ≥ 18 months. This is consistent with prior reports identifying *MGMT* methylation as a positive and independent predictor of improved survival in patients with GBM [42,43]. GBM tumors with *MGMT* methylation have been shown to have increased susceptibility to temozolomide, the standard of care chemotherapeutic agent [44]. However, a meta-analysis of 10 eligible studies concluded that, although *MGMT* methylation was associated with longer overall survival, its use as an indicator of progression-free survival is less clear [43]. This could be due, in part, to the variable cut-off value that is used at various institutions to determine one’s *MGMT* methylation status [45].

Our findings are similar to those by Tixier et al. who also studied the synergistic value of adding radiomics to *MGMT* methylation status to predict survival [46]. In their study, a total of 8 out of 286 texture features investigated were identified with significant prognostic value in determination of survival. After combining radiomics and methylation status of MGMT, they showed that patients who had both MGMT methylation and high negative skewness of Gabor edge enhancement (from radiomic analysis) had a longer median overall survival of 22.7 months vs. 12.2 months in patients without these features. 

Although the prognostic value of non-amplified *EGFR* and mutated *ATRX* in the prediction of longer survival in patients with GBM has been demonstrated previously [47,48], our study failed to show such an association. The exact reason for this discrepancy is not clear, however, it is plausible that small sample size and absence of data (e.g., *ATRX*) in a subset of patients (only available in 103/116, 89%) could have contributed to lack of significance.

Importantly, our study was validated with an external cohort consisting of patients from a different institution than the training cohort. In fact, the sensitivity of the model increased from 80.1% in the training cohort to 100% in the external cohort. Although a few other groups have shown the reproducibility of their radiogenomic findings in an external dataset [26,27], additional studies are needed to further improve the generalizability of radiomics for prognostic applications. 

Our study has several limitations. The retrospective nature introduces biases such as selection bias and patient recruitment through convenience sampling. A prospective approach can help to mitigate some of these limitations and allow for more even distribution of patients by demographic and histopathological variables. We only included treatment-naïve patients with GBM and, therefore, the results cannot be applied to patients with recurrent GBMs or to those who underwent treatment at other institutions. We only studied patients diagnosed with the most recent WHO classification of GBM: *IDH*-wildtype grade 4 astrocytoma. The results of our study and described predictive model cannot be applied to patients with *IDH*-mutant WHO grade 4 astrocytoma without further validation. Although our sample size is relatively small and data training was performed on imaging data from a single institution, the inclusion of an external validation cohort from a different institution increased the validity and generalizability of our predictive model. Furthermore, tumor heterogenicity may result in different biomarker results had an adjacent region of tumor been sampled instead for pathological analysis. A similar phenomenon has been documented using radiogenomic analysis, with MRI-defined radiologic habitats varying significantly among different patient survival groups [34]. Our study can also be limited by the variable reproducibility and reliability of radiomics [49]. A recent secondary analysis of two prospective studies of 48 total patients with GBM undergoing subsequent brain MRIs with no interval treatment demonstrated low repeatability of intensity and texture features, both of which depend on voxel intensity [49]. However, after data normalization, there was significant improvement in inter-scan repeatability [49]. Of note, even with normalization, Hoebel et al. found more repeatability for normalized FLAIR images than for normalized T1 post contrast images, likely due to the added variability of image acquisition timing after contrast injection. In our image analysis, we applied preprocessing image normalization to mitigate this limitation and maximize reproducibility.

## 5. Conclusions

In summary, we trained a multimodal radiogenomic model by combining age, *MGMT* status, and seven MRI texture features to predict longer (≥18 months) survival of patients with IDH-wildtype, WHO grade 4 GBM. Overall, testing this model with an external validation cohort yielded 89.3% accuracy with a sensitivity of 100%. If its potential is realized in a larger prospective study, this model can provide valuable prognostic information that can help guide management and support treatment decision making when consoling newly diagnosed patients and their families.

## Figures and Tables

**Table 1:** Demographic data and histopathological biomarkers in training cohorts.

	Total	Survival ≥ 18 Months (*n* = 42)	Survival < 18 Months (*n* = 74)	*p* Value
Age (mean ± SD)	59.6 ± 13.9	55.8 ± 14.6	61.7 ± 13.1	0.030
Sex (M/F)	62/54	21/21	41/33	0.575
Tumor side (right/left)	65/51	22/20	43/31	0.552
Tumor location (*n*/%)				0.266
Frontal lobe	45/39%	16/14%	29/25%	
Parietal lobe	10/9%	3/3%	7/6%	
Temporal lobe	48/41%	16/14%	32/27%	
Occipital lobe	5/4%	4/3%	1/1%	
Cerebellum	1/1%	1/1%	0/0%	
Basal ganglia	7/6%	2/2%	5/4%	
*MGMT* (methylated/non-methylated)	53/63	25/17	29/45	0.035
* *ATRX* (wildtype/mutated)	85/18	29/9	56/9	0.221
** *EGFR* (amplified/non-amplified)	74/41	25/17	49/24	0.414

* *ATRX* available in 103 patients; ** *EGFR* available in 115 patients.

**Table 2:** Diagnostic performances of independent classifiers and final radiogenomic model in prediction of survival in training and external validation datasets.

	Training Set (*n* = 116)	External Validation Set (*n* = 40)
	AUC/Sensitivity/Specificity	AUC/Sensitivity/Specificity
Age	0.64/61.9/67.6	0.70/91.7/42.9
MGMT status	0.60/59.5/60.8	0.80/75.0/85.7
Radiomic model (combined 7 textures)	0.71/69.0/70.3	0.76/91.7/60.7
* Radiogenomic model	0.77/81.0/66.0	0.89/100/78.6

* Radiogenomic model was constructed by combining age, *MGMT* status, and radiomic model.

## Data Availability

Data are contained within the article and Supplementary Materials.

## Supplementary Materials

The following supporting information can be downloaded at: https://www.mdpi.com/article/10.3390/cancers16030589/s1, Table S1: List of contributing MRI texture features following LASSO regularization and logistic regression analysis.
