# Supplementary material for: Multiparametric Radiogenomic Model to Predict Survival in Patients with Glioblastoma
Source: Cancers (Basel). 2024 Jan 30;16(3):589. doi: 10.3390/cancers16030589 (PMC10854536; doi:10.3390/cancers16030589)
Supplement: Supplementary file 1 [file cancers-16-00589-s001.zip › cancers-2815719-supplementary.pdf]

Supplementary Table S1: List of contributing MRI texture features following LASSO regularization and logistic regression analysis.

**Texture feature:**	**AUC /Sensitivity /Specificity /Threshold**
GLCM Informational Measurement Correlation 1	0.63 / 90.5 / 33.8 / 0.14
GLRLM Long Run Emphasis	0.60 / 78.6 / 43.2 / 1.51
GLRLM Run Percentage	0.60 / 71.4 / 48.6 / 0.89
GLSZM Zone Percentage	0.61 / 71.4 / 51.4 / 0.22
GLDMGL Dependence Non-Uniformity Norm	0.60 / 90.5 / 29.7 / 0.16
GLDM Dependence Entropy	0.61 / 57.1 / 62.2 / 8.0
GLCM Informational Measurement Correlation 1	0.62 / 45.2 / 81.1 / 0.18

The texture features in the top six rows are from FLAIR images. The texture feature in the bottom row is from ADC images. Key: GLCM (Gray level co-occurrence matrix), GLRLM (Gray level run length matrix), GLSZM (Gray level size zone matrix), GLDMGL (Gray level dependence matrix), GLDM (Gray level dependence matrix).
